# Dynamics of Changes in Isometric Strength and Muscle Imbalance in the Treatment of Women with Low back Pain

**DOI:** 10.1155/2020/6139535

**Published:** 2020-01-11

**Authors:** Jacek Wilczyński, Alicja Kasprzak

**Affiliations:** Laboratory of Posturology, Collegium Medicum, Jan Kochanowski University, Kielce, Poland

## Abstract

The aim of the study was to evaluate the dynamics of isometric changes in strength and muscular lumbar-pelvic imbalances in the treatment of women with low back pain. Forty-one women, nineteen in the study group (A) and twenty-two in the control group (B), participated in the study. Magnetic resonance imaging (MRI) was performed to assess the degree of degenerative changes in the lumbar spine. The diagnosis of isometric muscle strength and their imbalances was performed with the Tergumed 700 device. After six weeks of therapy in the study group (A), there was a significant improvement in the strength of all the examined muscle groups. However, in the control group (B), significant improvement occurred only in the strength of the lumbar flexor muscles and the flexor muscles on the left side. Furthermore, there was a significant intensification of the imbalance of left flexor muscle strength compared to right flexor strength in group B. Significant differences in favour of the study group (A) concerned the strength of the rotator muscles to the left, the strength of the extensor muscles of the lumbar spine, the strength of the flexors of the lumbar spine to the right, and the balance of the strength of the lumbar spine flexors to the left compared to the strength of the flexor muscles to the right. Therapy with the Tergumed 700 system leads to an increase in the muscle strength of the lumbar and pelvic complex, compensating for its imbalance, bringing beneficial effects in the treatment of low back pain.

## 1. Introduction

Low back pain is one of the most commonly diagnosed diseases of the osteoarticular system [1]. It is also the most frequently reported ailment [2] and the second main cause of sickness absence [3]. It is the most common cause of the inability to perform work [4] and is one of the main causes of physical disability of people below the age of 45 [5]. Pain syndromes can be divided into specific and nonspecific [6, 7]. Myofascial overloads, ligament injuries, and psychogenic factors are considered to be the causes of those nonspecific [8, 9]. Specific pains are most often caused by a herniated nucleus, spondylolisthesis, spinal canal stenosis, degenerative changes of the interappendix joints, vertebral fractures, spinal tumours, or inflammatory diseases [10, 11]. Pain sensations may be dull and diffuse but may also be shooting, stabbing, causing a burning or stinging sensation [12, 13]. In the population, nonspecific back pain is the most common, that is, the basis for which the specific pathology that causes the pain cannot be found [14, 15]. In acute pain, appearing for the first time in life, only 2% of patients can determine its cause [16, 17]. The incidence of this disease entity causes a significant burden on the state budget. The largest direct costs are generated by diagnostics, treatment, and rehabilitation, while indirect costs are disability pensions, benefits, and sick leaves at work [18, 19].

There are many suggestions in the available literature for conservative treatment of low back pain [20, 21]. Properly targeted kinesiotherapy plays a dominant role [22, 23]. In recent years, various devices have been developed to create optimal conditions for conducting isolated exercises regarding the lumbar region of the spine [24]. Tergumed is one such system used in the diagnosis and therapy of low back pain.

Research conducted using this system, however, generally took the short duration of therapy into account, which hindered its objective assessment. There is also a lack of research on the dynamics of changes in strength and muscle imbalance at individual periods of therapy, or comparison of results with the control group. Therefore, there was a need to conduct a comprehensive, objective, and controlled clinical study as well as a thorough analysis of the dynamics of changes in muscle activity and their imbalances [25].

For stabilization, the spine needs both muscle strength and stiffness, to which the muscles contribute. Due to the recognition of the close relationship between muscle function and low back pain, a new paradigm has been developed regarding the function and dysfunction of the deep muscle system. Moreover, the characteristics of exercises necessary for the rehabilitation of patients with low back pain have also been determined. This model has contributed to the modification of programmes for the rehabilitation of patients with this type of ailment by introducing rotation and extension exercises [26–28]. Basically, there are two main modes of action aimed at improving the protective function of muscles in relation to the spine joints. The first of them utilises the principle of minimising the forces affecting the lumbar spine during basic motor activity, and the second the optimal control of the lumbar-pelvic complex. Patients suffering from low back pain may demonstrate a lack of muscle tone normalization even after the pain subsides [29, 30]. Therefore, in this study, the authors focused on the assessment of isometric muscle strength and muscle balance [31].

The aim of the study was to assess the dynamics of changes in isometric strength and muscular imbalances of the lumbar and pelvic complex in the treatment of women with low back pain. The authors assumed that this therapy, by developing muscle strength and improving balance, improves the stabilization of the lumbar-pelvic complex and demonstrates beneficial effects in the treatment of people with low back pain.

## 2. Materials and Methods

Forty-one women aged 60–75 took part in the study (*X* = 65.3; SD = 6.5). They were patients of the Rehabilitation Clinic who were diagnosed with low back pain. The inclusion criteria were low back pain, degenerative changes of the lumbar spine visible in magnetic resonance imaging, age 60–75 years, patient's consent to participate in the study, and not undergoing lumbar spine rehabilitation with a different kinesiotherapy method than the one applied in the study at the time of research.

The exclusion criteria were less than 3 months from the onset of the acute discopathy phase, fresh fractures, short remission intervals in the course of rheumatic diseases, inflammatory diseases at the stage of exacerbation, for example, ankylosing spondylitis, hernia (abdominal, inguinal), osteoporosis with mineral density of bones up to 80% of the average for a given age, cancer, and spinal deformities.

Magnetic resonance imaging (MRI) was performed to assess the degree of degenerative changes in the lumbar spine. Randomly tested using a computer number generator, the subjects were assigned to two groups. The study group (A) comprised nineteen participants, while the control group (B) totalled twenty-two subjects.

In the study group (A), central stabilization exercises and therapy using the Tergumed 700 system were performed. In the control group (B), only central stabilization exercises were performed. The therapy lasted 6 weeks. All research procedures were carried out in accordance with the 1964 Declaration of Helsinki and with the consent of the Bioethics Committee at the Regional Medical Chamber in Kraków (Poland) No. 73/KBL/OIL/2016 from May 4, 2016.

### 2.1. Assessment of the Dynamics of Isometric Changes in Muscular Strength and Muscular Imbalances of the Lumbar-Pelvic Hip Complex

The muscle strength test of the lumbar-pelvic hip complex was performed using the Tergumed 700 system. This system was TÜV Süd 0123 certified and met the requirements of Directive 93/42 EEC [32]. The test was performed in flexion, extension, lateral flexion (left/right), and rotation (left/right). The test took place in a seated position. Each device was adapted to the patient. The authors made sure that the axis of motion was correct and that the subject was well stabilized. Isometric strength of the muscles was tested. The measurement was carried out using a built-in dynamometer (Nm). On each of the four Tergumed devices, a strength and muscle imbalance test was performed. One test repetition and two research repetitions were carried out, from which the average value was calculated. The examination was performed four times: before and after two, four, and six weeks of therapy.

#### 2.1.1. Central Stabilization Training

Central stabilization training was conducted on the basis of a scheme developed by Richardson et al. [33]. It consisted of three stages: training of local segmental control, of segmental control in a closed chain, and of segmental control in an open chain. A warm-up using cycloergometers and deep muscle activation in low positions was also performed. The ability to properly activate muscles was palpated, enabling the transition during training to exercises in higher positions. Coordination and balance exercises were carried out with the use of Swiss balls, aerodynamic discs, sphere segments, and elastic bands. In addition, exercises for stretching and relaxing contracted muscles were used. Central stabilization training in both groups (A and B) was conducted equally by the same physiotherapist and was applied for 30 minutes, 5 days a week, and for 6 weeks from May 2016 to March 2017.

#### 2.1.2. Therapy Using the Tergumed 700 System

Tergumed 700 is a line of 4 devices for diagnosis and therapy of the lumbar spine. Each device was responsible for 4 main directions of spinal movement (extension, flexion, lateral bend, and rotation). Before therapy, a maximum muscle strength test was performed on each device. Based on the test, an individual therapy plan using feedback was generated. The therapy was aimed at improving strength and compensating for muscular imbalances. The test also allowed for painless treatment. Thanks to programming the therapy based on the patient's current-condition test, it was a therapy that met the criteria of evidence-based medicine. The therapy followed the instructions given by Stevens [25, 34]. In the case of extension and flexion, 30–40% of maximum muscle work was used to activate the appropriate muscle groups. However, in terms of flexion and lateral flexion, this increased up to 60% of maximum muscle work. The loads gradually increased by 5% every 3 days. Patients initially performed 3 sets of exercises of 10 repetitions on each device. The number of repetitions was also gradually increased to 18 in the series. Therapy with the Tergumed 700 system was only carried out in the study group (A), for 1 hour a day, 5 days a week, and for 6 weeks, from May 2016 to March 2017.

### 2.2. Applied Statistical Methods

Analysis of variance (ANOVA) was used to assess the dynamics of isometric changes in strength and muscular imbalances. The above calculations were performed using the Statistica StatSoft computer program and the Microsoft Office Excel spreadsheet. Statistically significant differences were assumed for *p* < 0.05.

## 3. Results

In the study group (A), the average body height (cm) was (*X* = 162.65; SD = 5.86; *V* = 3.6), body mass totalled (kg) (*X* = 75.05; SD = 10.44; *V* = 13.90) and BMI equalled (kg/m^2^) (*X* = 28.43; SD = 4.14; *V* = 14.55). In the control group (B), the average body height (cm) was (*X* = 161.47; SD = 5.41; *V* = 3.35), body mass equalled (kg) (*X* = 73.05; SD = 15.11; *V* = 20.68) and BMI totalled (kg/m^2^) (*X* = 28.04 (kg/m^2^), SD = 5.88; *V* = 20.976). In both groups, the majority of subjects were overweight.

### 3.1. Dynamics of Changes in Muscular Strength and Muscular Imbalances of the Lumbar-Pelvic Hip Complex in the Study Group (A)

In the study group (A), the greatest absolute differentiation occurred for the strength of the extensor muscles of the lumbar spine, examined after 2 weeks of therapy (SD = 51.25), while the relative differentiation for rotator muscle strength to the left in the preliminary study was (*V* = 50.02 Nm) (Table 1). The largest absolute and relative differences in muscle balance concerned the ratio of flexor muscle strength to the left to flexor muscle strength to the right after 2 weeks of therapy (SD = 0.47, *V* = 42.21) (Table 1).

In the study group (A), there was a significant (*p* < 0.005) improvement in all examined muscle groups. After 6 weeks of therapy, the strength of the lumbar flexor muscles improved significantly (*p*=0.018) from an initial value of 61.54 Nm to 86.61 Nm (Table 2). The strength of the lumbar spine extensor muscles improved significantly (*p*=0.001) from an initial value of 105.85 Nm to 159.59 Nm (Table 2). Also, the rotation force to the left improved significantly (*p*=0.001), from the initial value of 26.65 Nm to the value of 46.005 Nm (Table 2). The strength of the muscles rotating the lumbar spine to the right improved significantly (*p*=0.001) from the initial value of 28.10 Nm to 46.5 Nm (Table 2). There was also a significant improvement in the strength of the lumbar flexor muscles to the left (*p*=0.039), from an initial value of 36.72 Nm to 53.71 Nm (Table 2). The right flexor muscles of the lumbar spine also improved significantly (*p*=0.049) from an initial value of 37.7 Nm to 52.07 Nm after 6 weeks of therapy (Table 2).

The balance of all tested lumbar-pelvic complex muscles was correct at individual measurement periods (Table 2). The strength of the antagonistic muscle groups was balanced.

### 3.2. Dynamics of Changes in Muscle Strength and Muscular Imbalances of the Lumbar-Pelvic Hip Complex in the Control Group (B)

In the control group (B), the greatest absolute differentiation was demonstrated by the strength of the lumbar extensor muscles examined after 2 weeks of therapy (SD = 53.69), while relative differentiation, rotator muscle strength to the right of the lumbar region, and preliminary examination totalled (*V* = 62.23) (Table 3).

The greatest absolute differentiation of muscle balance concerned the ratio of rotator muscle strength to the left compared with rotator muscle strength to the right, examined after 6 weeks (SD = 1.84). Relative differentiation, however, concerned the ratio of rotator muscle strength to the left compared to the strength of rotator muscle to the right, examined after 4 weeks of therapy (*V* = 395.18) (Table 3).

Flexor muscle strength improved significantly (*p*=0.002) from an initial value of 50.37 Nm to 74.06 Nm after 4 weeks, only to slightly deteriorate after 6 weeks of therapy to 73.83 Nm (Table 2). The strength of the flexor muscles to the left improved significantly (*p*=0.030) from 29.27 Nm to 46.8 Nm after 6 weeks of therapy (Table 2).

There was a significant (*p*=0.010) worsening of the left flexor muscle balance compared to right flexor strength after 6 weeks of therapy. The strength of both of these muscle groups did not balance (Table 2).

### 3.3. Comparison of the Dynamics of Changes in Muscle Strength and Muscular Imbalances of the Lumbar-Pelvic Hip Complex in Groups A and B

Before therapy, muscle strength and their imbalance did not show significant differences between groups A and B.

After 2 weeks of therapy, there were significant (*p*=0.03) differences in the strength of the rotator muscles to the left of the lumbar region (Nm). In group A, there was greater improvement in muscle strength responsible for spinal rotation to the left (40.2 Nm) compared to the control group (28.28 Nm) (Table 4).

After 4 weeks of therapy, a significantly (*p*=0.03) better result was observed in the study group (A) compared to the control group (B) in the strength of the left rotator muscles. The test group (A) achieved an average result of 42.353 Nm and the control group (B) 31.42 Nm (Table 4). In addition, a significantly (*p*=0.01) better result was observed in the strength of the muscles rotating the lumbar spine to the right in group A. In the study group (A), this force was 45.43 Nm and in the control group (B), 31.82 Nm (Table 4).

After 6 weeks of therapy, the difference between groups in left rotator muscle strength remained (*p*=0.02) in favour of the study group (A). On average, in the study group (A), the result was 46.01 Nm and in the control group (B), 33.1 Nm (Table 4). This also applies to extensor muscle strength (*p*=0.02), which in the study group (A) increased to 159.6 Nm compared to the result in the control group (B) of 121.68 Nm (Table 4). There was also a significant (*p*=0.02) difference in lumbar spine flexor muscle strength to the right in favour of the study group. In the study group (A), the average value of this force was 52.07 Nm and in the control group (B), 38.92 Nm (Table 4).

A significant (*p*=0.03) difference was also observed in the balance of lumbar flexor muscle strength to the left compared with the right flexor muscle strength. Imbalance in the study group (A) remained within normal limits (1.05 on average), while in the control group (B), an abnormal increase in the strength of the flexor muscles to the left was observed compared to the flexors to the right (average 1.21) (Table 4).

## 4. Discussion

In the treatment of low back pain, it is of key importance to know the cause of the ailment and focus therapy on the problem occurring in a given patient. In developed societies, we are increasingly dealing with people who work long hours every day in a seated position [35]. In the case of weakening of the lumbar-pelvic hip complex muscles or their large imbalance, the rapid onset or asymmetrical lifting of a small weight can result in unilateral overload, muscle spasm, and trauma to the spine. This is because a sedentary lifestyle results in a loss of muscle mass and a gradual, systematic decrease in strength and flexibility.

Most studies available in the literature regarding physiotherapy in low back pain assessing individual methods or therapies are based on the evaluation of the training programme. Patients are examined before and after therapy, and in some cases, additionally in the middle of the therapy [36]. In this study, the authors evaluated the dynamics of changes in the strength of the lumbar-pelvic hip complex muscles and the equalization of their imbalances 4 times: before therapy, and after 2, 4, and 6 weeks of its duration.

Many authors have evaluated the effects of treating low back pain similarly as in the authors' research. Pranata et al. [37] investigated the coordination of lumbar extensor muscle work in patients with low back pain. Biofeedback was used in the form of a sinusoid, on which the indicator of the isometric force with which the patient exercised moved. Maximum values during the exercise oscillated between 20% and 50% of the patient's maximum isometric strength. A much smaller degree of sinusoid mapping (both voltage increase and relaxation) was observed in comparison to the control group without low back pain. There were also correlations between the increase in sinusoidal mapping during the return to the starting position and the increase in disability measured by the Oswestry questionnaire. In these studies, it has been shown that the control of extensor muscles in the lumbar region is impaired among patients with low back pain. Training using the Tergumed 700 system applied by the authors of this study was also based on biofeedback in the form of a sinusoid, which, as it has been shown in studies, has a positive effect on impaired muscle coordination.

França et al. [29] compared central stabilization training with strength training in fighting back pain, reducing disability and activating the transverse abdominal muscle. Both types of training gave satisfactory results, but central stabilization training proved to be more effective, mainly in the area of transverse abdominal muscle activation. However, as stated by Stevens et al. [38], strength-coordination training with the use of the Tergumed 700 system also activates the multifunctional muscle, especially when loaded with 30% of the maximum extension force. In these studies, it has been shown that strength training also affects deep muscle activity. In the research conducted by Parkkola et al. [39], it was also indicated that patients with low back pain had weakened muscles compared to the control group, as demonstrated by the isometric force test. Ruas and Viera [40] conducted a study on muscle strength and imbalance in low back pain, the results of which demonstrated that imbalance, mainly of flexor muscle strength relative to extensor muscle strength, may be associated with chronic lumbar spine pain.

Although pain syndromes are a complex and multifactorial problem, many authors associate them with muscle weakness [41]. As reported by Rossi et al. [42], therapy of back pain syndromes should include training of the efficiency and strength of muscles, mainly of the extensors. As reported by Steele et al. [43], such training should be conducted with the pelvis stabilized so as to exclude the involvement of other muscles, for example, the hip extensors. From the research by Catala et al. [44], it may be assumed that dorsal muscle training is beneficial in reducing lumbar pain among patients with low back pain. Patients who experience lumbar pain due to lumbar pain syndrome have reduced strength in their trunk muscles, mainly the extensors [44]. The legitimacy of the authors' research is also confirmed by other authors. Wang et al. [45] conducted a study regarding the impact of a 12-week standardized training programme on patients with low back pain. The results of the study showed significant improvement in muscular strength as well as compensation of flexion and extensor muscles of the lumbar region. In this study, the positive effect of training using the Tergumed system on muscle strength has been exhibited. However, a disadvantage of this study was the lack of precise specification of the group of subjects. Haag et al. [46] used the Tergumed system in their research to assess the strength of the dorsal muscles in athletes complaining of and not reporting pain in the lumbar spine. In the second group without pain, significantly higher isometric strength of the trunk muscles was observed. Nitera-Kowalik et al. [47] conducted studies on the impact of comprehensive therapy using the Tergumed system on improving coordination, compensating for muscular imbalances, the degree of disability caused by low back pain, and reducing pain sensations in patients treated at sanatoriums. Improvement in muscle imbalances was observed here for all of the examined muscle groups. However, this study did not include a control group.

In this study, in group A, in which the Tergumed 700 system was additionally used, there was a significant improvement in the strength of all the examined muscle groups after 6 weeks of therapy. Increased muscle strength responsible for extension of the lumbar spine and rotation to the left occurred after 2 weeks. This maintained after 4 and 6 weeks of therapy. The strength of the muscles rotating clockwise improved after 4 weeks and was maintained after 6 weeks of therapy. On the other hand, the strength of the trunk flexor muscles and those responsible for lateral flexion improved in the final measurement period. This suggests that the 6-week training programme is optimal for achieving strength improvement in all of the examined muscle groups.

The definitely worse results obtained in muscle strength in the control group (B) suggest that traditional central stabilization training has less of an effect on muscle strength. The differences between groups A and B became apparent after 2 weeks of therapy in terms of the force of rotation to the left. Then, after 4 and 6 weeks of therapy, the difference concerned the strength of both-sided rotation, extension, and flexion to the right. Muscle imbalance of the lumbar-pelvis-hip complex in the study group (A) remained within normal limits (1.05 on average), while in the control group (B), an abnormal increase in the strength of the flexor muscles to the left compared with the flexors to the right (average 1.21) was observed.

Therapy using the Tergumed system, through its programming based on objective patient examination, is effective in treating low back pain. Its use is also supported by economic considerations because one therapist can simultaneously rehabilitate 4 patients according to individual programmes. It is also important that objective examination before and after therapy allows verification of the applied therapeutic programme.

## 5. Conclusions

After 6 weeks of therapy in the study group (A), there was a significant improvement in the strength of all examined muscle groups. However, in the control group (B), significant improvement only occurred in the strength of the lumbar flexor and flexor muscles on the left side. In addition, in group B, there was significant deterioration of imbalance regarding the left flexor muscle strength compared to the right flexor strength. Significant differences in favour of the study group (A) concerned the strength of the rotator muscles to the left, the strength of the extensor muscles of the lumbar spine, the strength of the flexors of the lumbar spine to the right, and the balance of strength of the flexors of the lumbar spine to the left compared with the strength of the flexor muscles to the right. Therapy with the Tergumed 700 system leads to an increase in the muscle strength of the lumbar-pelvic complex and compensation for its imbalance, which provides beneficial effects in the treatment of low back pain.

## Figures and Tables

**Table 1:** Variables of muscle strength and their imbalance in the study group (A).

Variable	Prelim-test *X*; SD	Prelim-test CV	Test after 2 weeks *X*; SD	Test after 2 weeks CV	Test after 4 weeks *X*; SD	Test after 4 weeks CV	Test after 6 weeks *X*; SD	Test after 6 weeks CV
Strength of flexor muscles in lumbar spine section	61.54 ± 19.05	30.95	76.85 ± 29.94	38.96	81.23 ± 24.43	30.08	86.61 ± 24.79	28.63
Strength of extensor muscles in lumbar spine section	105.82 ± 28.45	26.88	142.71 ± 51.25	35.91	154.97 ± 37.87	24.44	159.6 ± 45.02	28.21
Strength of rotator muscles in left direction of lumbar spine section	26.65 ± 13.33	50.02	40.2 ± 16.64	41.4	42.35 ± 12.93	30.52	46.01 ± 16.04	34.88
Strength of rotator muscles in right direction of lumbar spine section	28.1 ± 10.45	37.18	37.3 ± 14.25	38.2	45.43 ± 14.38	31.65	46.5 ± 18.5	39.79
Strength of flexor muscles in left direction of lumbar spine section	36.72 ± 17.8	48.47	45.16 ± 19.68	43.58	50.63 ± 20.2	39.89	53.71 ± 17.88	33.3
Strength of flexor muscles in right direction of lumbar spine section	37.7 ± 14.5	38.48	42.72 ± 16.55	38.73	50.44 ± 20.36	40.37	52.07 ± 18.81	36.12
Ratio of flexor to extensor muscle strength	0.59 ± 0.14	23.09	0.56 ± 0.16	29.29	0.55 ± 0.18	32.36	0.56 ± 0.15	26.01
Ratio of rotator muscle strength in left direction to rotator muscle strength in right direction	0.94 ± 0.23	24.82	1.12 ± 0.36	32.37	0.91 ± 0.25	27.59	1.04 ± 0.27	25.72
Ratio of flexor muscle strength in left direction to flexor muscle strength in right direction	0.97 ± 0.23	23.99	1.1 ± 0.47	42.21	1.04 ± 0.22	21.54	1.05 ± 0.24	22.56

**Table 2:** Differences in muscle strength and imbalance before and after 6 weeks of therapy in the study group (A) and the control group (B) demonstrated by analysis of variance (ANOVA).

Variable	*F*	*p*
*Study group (A)*
Strength of flexor muscles in lumbar section	3.57	**0.018**
Strength of extensor muscles in lumbar section	6.54	**0.001**
Strength of rotator muscles in left direction of lumbar section	6.16	**0.001**
Strength of rotator muscles in right direction of lumbar section	6.44	**0.001**
Strength of flexor muscles in left direction of lumbar section	2.94	**0.039**
Strength of flexor muscles in right direction of lumbar section	2.75	**0.049**

*Control group (B)*
Strength of flexor muscles in lumbar section	5.34	**0.002**
Strength of flexor muscles in left direction of lumbar section	3.13	**0.030**
Ratio of flexor muscle strength in left direction to flexor muscle strength in right direction	4.05	**0.010**

**Table 3:** Variables of muscular strength and their imbalance in the control group (B).

Variable	Prelim-test *X*; SD	Prelim-test CV	Test after 2 weeks *X*; SD	Test after 2 weeks CV	Test after 4 weeks *X*; SD	Test after 4 weeks CV	Test after 6 weeks *X*; SD	Test after 6 weeks CV
Strength of flexor muscles in lumbar spine section	50.38 ± 23.24	46.13	67.81 ± 22.95	33.84	74.06 ± 20.38	27.51	73.83 ± 23.74	32.16
Strength of extensor muscles in lumbar spine section	93.59 ± 43.66	46.66	120.06 ± 53.69	44.72	129.42 ± 46.43	35.88	121.68 ± 51.57	42.38
Strength of rotator muscles in left direction of lumbar spine section	20.88 ± 12.99	62.23	28.28 ± 16.2	57.29	31.42 ± 17.28	54.99	33.1 ± 17.99	54.36
Strength of rotator muscles in right direction of lumbar spine section	24.03 ± 14.86	61.84	32.62 ± 18.89	57.92	31.82 ± 16.28	51.17	37.53 ± 20.53	54.71
Strength of flexor muscles in left direction of lumbar spine section	29.26 ± 17.79	60.79	37.52 ± 19.43	51.77	41.78 ± 20.46	48.98	46.8 ± 20.89	44.65
Strength of flexor muscles in right direction of lumbar spine section	30.01 ± 15.17	50.56	36.11 ± 15.49	42.91	39.3 ± 16.12	41.02	38.92 ± 16.44	42.23
Ratio of flexor to extensor muscle strength	0.57 ± 0.23	40.63	0.62 ± 0.18	28.58	0.6 ± 0.15	24.51	0.7 ± 0.32	45.39
Ratio of rotator muscle strength in left direction to rotator muscle strength in right direction	0.88 ± 0.34	38.51	1.01 ± 0.59	58.06	6.8 ± 0.85	395.18	1.28 ± 1.84	143.5
Ratio of flexor muscle strength in left direction to flexor muscle strength in right direction	0.97 ± 0.23	23.24	1.01 ± 0.25	24.79	1.08 ± 0.28	25.94	1.21 ± 0.21	17.02

**Table 4:** Differences in the dynamics of changes in muscle strength and their imbalance between the study group (A) and the control group (B) demonstrated by analysis of variance (ANOVA).

	Variable	Group A (Nm) *X*; SD	Group B (Nm) *X*; SD	*F*	*p*
Differences between study (A) and control (B) groups after 2 weeks of therapy	Rotator muscle strength in left direction of lumbar section	40.2 ± 16.64	28.28 ± 16.2	5.38	**0.03**

Differences between study group (A) and control group (B) after 4 weeks of therapy	Rotator muscle strength in left direction of lumbar section	42.35 ± 12.93	31.41 ± 17.28	5.13	**0.03**
	Rotator muscle strength in right direction of lumbar section	45.43 ± 14.38	31.82 ± 16.28	7.93	**0.01**

Differences between study group (A) and control group (B) after 6 weeks of therapy	Strength of extensor muscles in lumbar spine section	159.59 ± 45.02	121.68 ± 51.59	6.19	**0.02**
	Rotator muscle strength in left direction of lumbar section	46.01 ± 18.04	33.10 ± 18	5.79	**0.02**
	Extensor muscle strength in right direction of lumbar section	52.07 ± 18.81	38.90 ± 16.44	5.71	**0.02**
	Ratio of flexor muscle strength in left direction to flexor muscle strength in right direction	1.05 ± 0.24	1.21 ± 0.21	4.99	**0.03**

## Data Availability

The data and materials supporting the conclusions of this article are included within the article.
